# [Retracted] MicroRNA-124-3p inhibits the growth and metastasis of nasopharyngeal carcinoma cells by targeting STAT3

**DOI:** 10.3892/or.2024.8807

**Published:** 2024-09-02

**Authors:** Shan Xu, Ning Zhao, Lian Hui, Min Song, Zi-Wei Miao, Xue-Jun Jiang

Oncol Rep 35: 1385–1394, 2016; DOI: 10.3892/or.2015.4524

Following the publication of this paper, it was drawn to the Editor's attention by a concerned reader that certain of the cell apoptotic data in Fig. 4 on p. 1389 and the migration and invasion assay data shown in Figs. 6 and 7 on p. 1391 were strikingly similar to data that were submitted for publication at around the same time in different articles written by different authors at different research institutes (several of which have subsequently been retracted). In addition, there appeared to be instances of duplication of the same data within Figs. 7 and 8, where data that were intending to have shown the results from differently performed experiments had apparently been derived from the same original sources. Owing to the fact that the contentious data in the above article had already been submitted for publication elsewhere prior to its submission to *Oncology Reports*, the Editor has decided that this paper should be retracted from the Journal. The authors were asked for an explanation to account for these concerns, but the Editorial Office did not receive a reply. The Editor apologizes to the readership for any inconvenience caused.

